# Determination of Virulence Factors and Resistance Profile of Methicillin-Resistant *Staphylococcus aureus* Strains among Different Types of *spa*, *agr*, and SCC*mec*

**DOI:** 10.1155/2022/5863310

**Published:** 2022-10-15

**Authors:** Mohammad Latifpour, Tahmineh Narimani, Amin Sadeghi, Mohammad Niakan

**Affiliations:** ^1^Department of Microbiology, Faculty of Medicine, Shahed University, Tehran, Iran; ^2^Department of Microbiology, School of Medicine, Isfahan University of Medical Sciences, Isfahan, Iran

## Abstract

In order to restrict the spread of methicillin-resistant *S. aureus* (MRSA) in hospitals, it is necessary to characterize isolates rapidly and precisely. The objective of this study was to determine virulence factors and resistance profiles of MRSA strains among *spa*, *agr*, and SCC*mec* types. In total, 55 MRSA isolates were collected from clinical specimens. The MRSA isolates were characterized by antimicrobial susceptibility testing, virulence genes, *agr* typing, *spa* typing, and SCC*mec* typing. According to our findings, all MRSA strains were resistant to cefoxitin; 88% and 86.7% of which were resistant to erythromycin and clindamycin, respectively. Type II *agr* was predominant with 54.54% frequency. Among 27 different *spa* types, type t030 was most frequently (25.45%). Most MRSA isolates (63.3%) were SCC*mec* type III. The *pvl* and *tst* genes were found in 25.3% and 32.7% of MRSA isolates, respectively. Among the MRSA strains, *ermA*, *ermB*, and *ermC* were present in 50%, 33.3%, and 57.3% of cases, respectively. In addition, 43 of the 55 MRSA strains (78%) harbored aminoglycoside resistance genes. The results of our study revealed that the MRSA rate in our region is dramatically high. Better infection control guidelines in hospitals, as well as ongoing epidemiological surveillance studies, could be strongly suggested for effective prevention of the spread of MRSA to inpatients.

## 1. Introduction

Among human pathogens, *Staphylococcus aureus* (*S. aureus*) is one of the most common ones [1]. Methicillin-resistant *S. aureus* (MRSA) strains have emerged as a major problem in hospitals, owing to the increased mortality rate associated with some of these infections. MRSA strain outbreaks have a significant impact on morbidity, mortality, and healthcare costs [2–4]. MRSA strains express virulence factors that play a key role in infection progression. The accessory gene regulator (*agr*) system regulates the expression of numerous virulence factors in *S. aureus*, and four major *agr* types have been identified to date. Different *agr* types have different properties and distributions in various geographic regions; thus, identification of the predominant types in each location would be of benefit [5, 6].

It is clear that the spread of MRSA around the world is constantly evolving, with new strains emerging in a variety of geographical regions. The *mecA* gene, which confers beta-lactam resistance, is found on the staphylococcal cassette chromosome mec (*SCCmec*) of MRSA strains. MRSA is divided into distinct epidemiological types based on the presence of the SCC*mec* element. The SCC*mec* typing method can help distinguish community-associated MRSA (CA-MRSA) from hospital-acquired MRSA (HA-MRSA) infections [7].

Continuous MRSA surveillance in each location necessitates monitoring the epidemiology, host characteristics, and transmission routes of emerging strains [8]. Therefore, clinicians must have a thorough understanding of MRSA's molecular epidemiology in order to assess the efficiency of preventative strategies and provide effective prophylaxis [9]. Prevention of MRSA transmission by screening patients, personnel, and the environment is a critical goal of infection control [9]. However, investigating the origins and routes of transmission of MRSA is possible only through the use of typing approaches, which are necessary for genetic characterization. There is a variety of molecular epidemiological methods used for MRSA surveillance, including multilocus sequencing typing (MLST), pulsed-field gel electrophoresis (PFGE), staphylococcal protein A (*spa*) gene sequencing, *agr*, and SCC*mec* typing [10]. Although each of these methods has a pretty good discriminating power, it has been demonstrated that combining genotyping methods is beneficial and advantageous for distinguishing distinct MRSA clones.

*spa* typing is a valuable typing instrument owing to its ease of use, cost-effectiveness, and uniform nomenclature, which is based solely on assessment of the repetition space in the X region of the *spa* gene [11]. The X region polymorphism, which encodes a part of the *spa* protein, is characterized by variations in tandem repeats as well as variations in base sequences within repetitions. In other words, in any strain of *S. aureus*, each motif consists of 24 base pairs, which are referred to as unique sequence motif repeats. The order of the repeats determines the *spa* type for a strain [12]. The *spa* types are important for identifying *S. aureus* outbreak isolates and infection control policies around the world. Over the last decade, studies have been conducted on the distribution of *spa*, *agr*, and SCC*mec* types in various geographic areas [13, 14]. Therefore, the current research is aimed at determining virulence and antimicrobial resistance profiles of MRSA isolates using *spa*, *agr*, and SCC*mec* typing.

## 2. Materials and Methods

### 2.1. Ethical Considerations

Ethics approval to perform this study was obtained from the institutional review board of Shahed University of Medical Sciences, Tehran, Iran (http://ethics.research.ac.ir/IR.SHAHED.REC.1398.089).

### 2.2. Detection and Isolation of MRSA

In this cross-sectional study, out of a total of 142 *S. aureus* isolates, 55 MRSA isolates were identified and included in this study, while the remaining isolates were excluded. The isolates were obtained from clinical samples including blood, urine, wounds, and cerebrospinal fluid collected from different wards (emergency, men, women, children, and intensive care unit) in Al-Zahra Hospital in Isfahan (Iran), then referred to the hospital laboratory. The *S. aureus*was identified using growth on mannitol salt agar, showing beta-hemolysis on 5% sheep blood agar, and being gram positive as well as producing catalase, coagulase, and DNase. The presence of the *nucA* gene was confirmed by PCR in all *S. aureus* isolates (Table 1).

### 2.3. Antimicrobial Susceptibility Testing and Detection of MRSA

The following antibiotics were tested for antibiotic susceptibility using the disk diffusion technique on Mueller-Hinton agar, and the results were recorded after incubation for 18 hours at 37°C and in accordance with the CLSI guidelines [15]: penicillin (10 *μ*g), erythromycin (15 *μ*g), gentamicin (10 *μ*g), tetracycline (30 *μ*g), clindamycin (2 *μ*g), rifampicin (5 *μ*g), cefoxitin (30 *μ*g), linezolid (5 *μ*g), and trimethoprim-sulfamethoxazole (5 *μ*g) (Mast, Merseyside, UK). The presence of the 310-base pair (bp) PCR product of the *mecA* gene was examined in all *S. aureus* isolates (Table 1), and cefoxitin (30 *μ*g) discs on Muller-Hinton agar plates were used to screen for MRSA isolates.

### 2.4. Genomic DNA Extraction

A DNA Mini Kit (Qiagen GmbH, Hilden, Germany) was used for genomic DNA extraction. Fresh colonies harvested from agar plates were washed with 500 *μ*l TE 1x and centrifuged for 10 minutes at 5000 rpm according to manufacturer's protocol. A suspension was then prepared in 200 *μ*l TE 1x with 20 *μ*l lysostaphin (200 *μ*g/ml final concentration) and incubated at 37°C for 20 minutes. Finally, the obtained DNA was dissolved 50 *μ*l RNase-DNase-free water (Sigma). DNA concentration was measured with a spectrophotometer.

### 2.5. SCC*mec* Typing

As described previously, multiplex PCR was used to identify various MRSA isolates using genomic DNA as the template [16]. The amplification started with a 3-minute denaturation step at 94°C, then 35 cycles of 30 seconds at 94°C, 1 minute at 55°C, 1 minute at 72°C, and finally 5 minutes at 72°C for final extension.

### 2.6. Detection of Virulence and Resistance Genes

To detect virulence genes such as hemolysin A (hla), toxic shock syndrome toxin (tst), staphylococcal enterotoxins (sea, seb, and sec), and Panton-Valentine leukocidin, PCR was used (pvl). As previously described, PCR assays were used to investigate the common aminoglycoside resistance genes (aac (6′)-aph (2^″^), aph3, ant4) and macrolide resistance genes (ermA, ermB, ermC) [17, 18].

### 2.7. Detection of *agr* Types

Multiplex PCR was performed to detect *agr* types using a set of primers containing a common forward primer (Pan) and reverse primer (*agrI, agrII, agrIII*, and *agrIV*) that are unique to each *agr* group [19]. The primer sequences are shown in Table 1.

### 2.8. Detection of *spa* Types

The identified MRSA strains were subjected to PCR to detect the *spa* gene (Table 1). The amplification reaction consisted of an initial denaturation step at 94°C for 5 min followed by 35 cycles of denaturation at 94°C for 40 second, hybridization at 56°C for 40 second, and extension to 72°C for 50 second, followed by final extension to 72°C for 5 minutes [11]. Sequencing was then performed on the PCR products. Also, after sequencing, the spa database server (http://spaserver.ridom.de/) was used to determine different types.

### 2.9. Statistical Analysis

For statistical analysis, SPSS Statistics 22.0 for Windows was used. Data were presented using descriptive statistics (frequency, percentage, mean, and standard deviation).

## 3. Results

### 3.1. Detection and Isolation of MRSA

In this study, 55 clinical MRSA isolates were recovered from blood samples (*n* = 12; 21.83%), nasal (*n* = 14; 24.45%), urine (*n* = 7; 12.72%), trachea (*n* = 8; 14.54%), wound (*n* = 12; 21.83%), and synovial (*n* = 2; 3.63%). According to our data, the nasal specimen has the highest frequency of MRSA (26%). The patients were divided into 29 (52%) males and 26 (48%) females. Participants in the study ranged in age from 9 to 86. Most of the study participants were in the 21–60-year-old group (66%).

### 3.2. Antibiotic Susceptibility Tests

Antibiotic susceptibility testing was performed on all MRSA isolates. All were resistant to cefoxitin and penicillin; 88% and 86.7% of them were resistant to erythromycin and clindamycin, respectively. On the other hand, all MRSA isolates were sensitive to linezolid (Table 2).

### 3.3. *agr* Typing

According to *agr* typing, 55 of the MRSA isolates belonged to one of agr types I, II, III, or IV. By using the *agr* typing method, 29.09% (*n* = 16), 54.54% (*n* = 30), 10.9% (*n* = 6), and 5.45% (*n* = 3) of isolates belonged to *agr* types I, II, III, and IV, respectively.

### 3.4. Prevalence of SCC*mec* Types, Virulence, and Resistance Genes

Most MRSA isolates (63.3%) were SCC*mec* type III. Also, the frequency of SCCmec types II and IX was 10.7% for each, 9.3% as SCC*mec* type V, 4% as SCC*mec* type I, and 2% as SCC*mec* type IV. In addition, 43 of the 55 MRSA strains (78%) harbored aminoglycoside resistance genes, with the presence of *aac(6*′*)-aph(2*^″^), *aph3*, and *ant4* genes among MRSA isolates, were 54%, 32.7%, and 31.3%, respectively. Among the 55 MRSA strains, macrolide resistance genes *ermC*, *ermA*, and *ermB* were detected in 35 (63.6%), 11 (20%), and 9 (16.4%) isolates, respectively. In our study, genes encoding staphylococcal enterotoxins *sea*, *seb*, and *sec* were found in 48%, 25.5%, and 12% of MRSA isolates, respectively. In addition, the *pvl* and *tst* genes were found in 25.3% and 32.7% of MRSA isolates, respectively.

### 3.5. *spa* Typing

Twenty-seven *spa* types were observed in this study. There were 19 *spa* types that were found only once in all of the 55 strains analyzed. Accordingly, single types of *spa* are extremely important in MRSA strains.  Table 3 shows that t030 and t037 predominate in clinical samples, especially in blood and nasal samples. As well as, phenotypic and genotypic traits of all our isolates are presented in Table 4 [21].

## 4. Discussion

MRSA strains are one of the leading causes of infections in hospitals, but infections from community-related MRSA have become a global public health threat over the recent decades [22]. The widespread occurrence of multidrug-resistant (MDR) MRSA augments the cost of antibiotic therapy and limits treatment options. During the last two decades, the widespread use of beta-lactam antibiotics in Iranian hospitals and medical facilities led to increased resistance to these antibiotics [23]. The results of the current study revealed that MRSA strains are resistant to erythromycin (88%), clindamycin (86.6%), tetracycline (68%), rifampicin (57%), and gentamicin (54.6%). Based on these results, linezolid was the most effective drug for MRSA in the study area. In addition, more than 93% of our MRSA isolates were MDR. We did not report the frequency of MRSA strains since the selection criteria of our study were isolation of MRSA strains, and methicillin-sensitive *S. aureus* (MSSA) strains were not included. As a protein synthesis inhibitor, erythromycin is widely utilized for the treatment of staphylococcal infections [24]. According to Mahdiyoun et al., the frequency of MRSA resistance tested for erythromycin was 84.4% [25]. A study conducted in Taiwan reported that the resistance rates for erythromycin and clindamycin were 94.9% and 86.5%, respectively [26]. High frequency of resistance to erythromycin and clindamycin antibiotics in the present study was in consistent with the findings of previous research in Iran [24] and India [27].

We also found SCCmec type III in a high percentage of MRSA isolates. Similarly, studies in Iran and other Asian countries have reported the high prevalence of SCC*mec* type III [28–30]. In MRSA isolates, SCC*mec* mobile genetic factor leads to an expansion of antibiotic resistance determinants as well as virulence factors, which can act as a large reservoir of resistance genes, enterotoxins, and other virulence factor genes. Our results showed that among MRSA with SCC*mec* type III, *sea*, *hla*, and *seb* were the most frequently found genes encoding virulence factors. Herein, the majority of the isolates with type III were resistant to erythromycin and clindamycin (55% and 54%) while all the isolates were resistant to gentamicin. The SCCmec typing has also been done in other parts of Iran in accordance with our study. A study by Ebrahim-Saraie et al., Moshtagheian et al., and Parhizgari et al. reported that SCCmec type IV dominated MRSA isolates; however, in line with our findings, most studies conducted in Iran described SCCmec type III as the predominant SCCmec type [31–33]. In the present study, among MRSA strains, *ermC* (63.6%) was the most commonly detected macrolide resistance gene, followed by *ermA* (20%) and *ermB* (16.4%). Also, the most frequently identified aminoglycoside resistance gene was *aac (6*′*)-aph (2*^″^) (54%). These findings are in contrary with the study by Hau et al. [34] conducted with clinical MRSA in the United States in which an incidence of 91.5% for ermA and 12.7% for *aac (6*′*)-aph (2*^″^) was reported. A similar finding was also reported by Yılmaz and Aslantaş [35] that the *ermC* and *aac (6*′*)-aph (2*^″^) genes were detected in 91.5% and 50% of *S. aureus* isolates, respectively.

In view of the widespread of MRSA isolates, it is imperative that the treating physician encourages the preservation of glycopeptides and linezolid only in the case of MRSA. In Iran, a major MRSA-associated problem is the result of increased incidence and hospitalization rates. Therefore, for screening, epidemiology, surveillance, and infection control, rapid and accurate typing of MRSA isolates is crucial [23]. Several genotyping techniques are available for identifying *S. aureus* strains in epidemiological studies. Sequence-based typing methods, such as MLST and *spa* typing, have several obvious advantages; for example, they are easily used, portable, reproducible, and able to provide comparable results compared to tape-based methods, such as small macrorestricted analysis [14]. One of the key regulators of *S. aureus*, which is involved in the regulation of bacterial virulence factors, is the *agr* system. There are currently four *agr* types identified in *S. aureus* strains (I, II, III, and IV). According to our study, the predominant agr type among the 55 MRSA isolates was type II, with a frequency of 54.54 percent. The majority (69.5%) of the isolates studied by Ghasemian et al., showed *agr* I, followed by *agr* III (30.5%) [36]. The *agr* type III is the most prevalent type of MRSA isolate, according to a study by Goudarzi et al., in Iran [37]. There is a significant relationship between *agr* types and specific pathogens [38], and the distribution of *agr* types varies by geographic region. The selected regions of the *spa* gene are usually short repeats of sequences with enough polymorphisms to allow isolated typing [20]. In the current work, 27 different *spa* types were found. *spa* typing analysis indicated that *spa* type t030 was the most common *spa* type found in 25.45% of isolates. The second most frequently identified *spa* type in our study was t037. These results are consistent with those of other studies in Iran and other Asian countries [10]. The *spa* type t037 was previously reported by Alreshidi et al. in Saudi Arabia [39], Chen et al. in China [40], and Goudarzi et al. in Iran [37]. In agreement with our study, in China, t030 was found to be one of the most common *spa* types (52.0%) of MRSA isolates [40]. We believed that t030 would result in longer bacterial survival and easier transmission. In this study, we reported that 7.27% of our isolates had *spa* type t325 and 19 *spa* types, which were detected, each, in one isolate.

## 5. Conclusion

According to recent studies, presence of these common SCC*mec* (III), *spa* (t030), and *agr* (II) types indicates that these MRSA strains are actively circulating in the healthcare setting of Isfahan Province. Despite the high diversity of our *spa* types (27/55) in this study, most of them are classified as t030 and t037 and are probably phylogenetically related. There is a possibility that these strains may spread to other parts of Iran or the world in relation to the tourism industry in Isfahan Province. Therefore, the current study emphasizes the importance of molecular typing in tracking global trends in the emergence, spread, and persistence of epidemic MRSA strains. Better infection control guidelines in hospitals, as well as ongoing epidemiological surveillance studies, could be strongly suggested for effective prevention of bacteria spread to inpatients and control nosocomial infections.

## Figures and Tables

**Table 1:** Oligonucleotide primers used in this study.

Primer	Sequence (5′-3′)	Amplicon size (bp)	References
*nucA* *nucA*	CTGGCATATGTATGGCAATTGTT TATTGACCTGAATCAGCGTTGTCT	613	[20]
*mecA* *mecA*	GATGAAATGACTGAACGTCCGATAA CCAATTCCACATTGTTTCGGTCTAA	310	[20]
*agr I* F *agr I* R	ATGCACATGGTGCACATGC GTCACAAGTACTATAAGCTGCGA	441	[19]
*agr II* F *agr II* R	ATGCACATGGTGCACATGC TATTACTAATTGAAAAGTGGCCATAGC	575	[19]
*agr III* F *agr III* R	ATGCACATGGTGCACATGC GTAATGTAATAGCTTGTATAATAATACCCAG	323	[19]
*agr IV* F *agr IV* R	ATGCACATGGTGCACATGC CGATAATGCCGTAATACCCG	659	[19]
*spa* F *spa* R	TAAAGACGATCCTTCGGTGAGC CAGCAGTAGTGCCGTTTGCTT	300-500	[11]

**Table 2:** Characteristics of antibiotic resistance pattern of MRSA and type of specimen. All results were expressed as percentages.

Type of specimen	ERY		CD		SXT		GM		TE		RA	
	S	R	S	R	S	R	S	R	S	R	S	R
Blood	1.3	20.0	0.7	20.7	9.3	12.0	10.7	10.7	7.3	14.0	8.7	12.7
Nasal	3.3	22.7	3.3	22.7	12.7	13.3	10.7	15.3	9.3	16.7	10.7	15.3
Urine	4.7	8.7	2.7	10.7	6.7	6.7	6.7	6.7	4.0	9.3	6.0	7.3
Trachea	0.7	14.0	2.0	12.7	8.0	6.7	2.0	12.7	4.0	10.7	4.0	10.7
Wound	1.3	20.0	4.7	16.7	10.7	10.7	13.3	8.0	6.0	15.3	11.3	10.0
Synovial	0.7	2.7	0.0	3.3	2.7	0.7	2.0	1.3	1.3	2.0	2.0	1.3
Total	12.0	88.0	13.3	86.7	50.0	50.0	45.3	54.7	32.0	68.0	42.7	57.3

ERY: erythromycin; CD: clindamycin; SXT: trimethoprim/sulfamethoxazole; GM: gentamicin; TE: tetracycline; RA: rifampin. All MRSA isolates were susceptible to linezolid, quinupristin/dalfopristin, and teicoplanin. S: sensitive; R: resistant. The frequency percentage was calculated according to the total number of MRSA isolates (55) not according to the number of isolates in each clinical sample. The data is presented as a percentage.

**Table 3:** Distribution of the *spa* types among different clinical samples.

Spa type	*N* (%) of isolates						Total *N* = 55
	Blood	Nasal	Urine	Trachea	Wound	Synovial	
t275	—	1 (100)	—	—	—	—	1
t4679	—	1 (100)	—	—	—	—	1
t7685	1 (50)	—	1 (50)	—	—	—	2
t3236	—	1 (100)	—	—	—	—	1
t790	—	1 (100)	—	—	—	—	1
t030	4 (28.6)	5 (35.7)	1(7.1)	2 (14.3)	2 (14.3)	—	14
t037	1 (12.5)	2 (25)	2 (25)	1 (12.5)	1 (12.5)	1 (12.5)	8
t3769	—	—	—	—	—	1 (100)	1
t3204	—	1 (50)	—	—	1 (50)	—	2
t314	—	—	—	—	1 (100)	—	1
t5163	—	—	—	—	1 (50)	1 (50)	2
t325	1 (25)	—	—	1 (25)	2 (50)	—	4
t1587	—	1 (100)	—	—		—	1
t223	—	1 (100)	—	—		—	1
t5593	—	—	1 (100)	—		—	1
t131	—	—	—	—	1 (100)	—	1
t15871	—	—	—	1 (100)		—	1
t159	—	—	—	1 (100)		—	1
t1360	—	—	—	—	2 (100)	—	2
t692	—	1 (100)	—	—	—	—	1
t2976	—	—	1 (100)	—	—	—	1
t2104	—	—	—	—	1 (100)	—	1
t1258	—	—	—	2 (100)	—	—	2
t1403	—	1 (100)	—	—	—	—	1
t2457	1 (100)	—	—	—	—	—	1
t3182	1 (100)	—	—	—	—	—	1
t459	1 (100)	—	—	—	—	—	1
Total	10 (18.2)	16 (29)	6 (11)	8 (14.55)	12 (21.8)	3 (5.45)	55

**Table 4:** Phenotypic and genotypic traits of all MRSA isolates in this study. Some data were collected with the study of Latifpour et al. [21].

Isolate number	*spa* type	*agr* type	Sample type	SCC*mec*	Virulence genes	Resistance profile	Resistance genes
1.	t030	II	Blood	III	*hla*, *sea*, *seb*	FOX, ERY, CD, SXT, GM, TE, RA	*aac* (*6*′)-*aph* (*2*^″^), *aph3*, *ermC*
2.	t030	II	Blood	III	*tst*	FOX, ERY, CD, SXT, GM, TE, RA	*aac* (*6*′)-*aph* (*2*^″^), *aph3*, *ermB*
3.	t030	IV	Wound	III	*pvl*, *hla*, *sea*, *seb*, *sec*	FOX, ERY, CD, SXT, GM, TE	*aac* (*6*′)-*aph* (*2*^″^), *ant4*, *ermC*
4.	t030	II	Trachea	III	*hly*, *sea*	FOX, ERY, CD, SXT, GM, RA	*aac* (*6*′)-*aph* (*2*^″^), *aph3*
5.	t030	I	Nasal	III	*hly*, *sea*	FOX, ERY, CD, SXT, GM, TE, RA	*aac* (*6*′)-*aph* (*2*^″^), *aph3*
6.	t030	II	Nasal	III	*pvl*, *tst*, *hly*, *sea*	FOX, ERY, CD, TE	*ermC*
7.	t030	II	Blood	III	*tst*	FOX, ERY, CD, SXT, GM, RA	*aph3*, *ermA*
8.	t030	I	Nasal	III	*hla*, *sea*	FOX, SXT, GM, TE, RA	*aph3*, *ant4*, *aac* (*6*′)-*aph* (*2*^″^)
9.	t030	II	Nasal	IX	*hla*	FOX, ERY, CD, SXT, GM, TE, RA	*ermC*
10.	t030	I	Nasal	V	*—*	FOX, GM	*ant4*, *aph3*, *ermA*
11.	t030	I	Blood	V	*pvl*, *hla*, *sea*	FOX, ERY, TE	*ermC*, *ermA*
12.	t030	IV	Wound	II	*hla*, *sea*, *seb*	FOX, ERY, CD, SXT, TE, RA	*ermC*, *ermB*
13.	t030	I	Trachea	II	*hla*, *tst*	FOX, ERY, TE	*ermC*, *ermB*
14.	t030	I	Urine	IX	*hla*, *tst*	FOX, ERY, CD	*ermC*, *ermA*
15.	t037	III	Urine	II	*hla*, *sec*	FOX, TE	*—*
16.	t037	I	Urine	III	*pvl*, *tst*, *hla*, *sea*, *seb*	FOX, ERY, CD, SXT, GM, TE, RA	*aac* (*6*′)-*aph* (*2*^″^), *ant4*, *aph3*
17.	t037	I	Nasal	III	*hla*	FOX, ERY, CD, SXT, GM, TE, RA	*aac* (*6*′)-*aph* (*2*^″^), *aph3*
18.	t037	III	Synovial	III	*hla*	FOX	*—*
19.	t037	II	Wound	III	*hla*, *sea*	FOX, TE	*—*
20.	t037	II	Nasal	IX	*pvl*, *tst*, *hla*, *sea*, *seb*	FOX, ERY, CD, SXT, GM, TE, RA	*aac* (*6*′)-*aph* (*2*^″^), *aph3*
21.	t037	I	Trachea	IX	*hla*, *sea*	FOX	*—*
22.	t037	I, II	Blood	IX	*hla*, *sea*	FOX, ERY, CD, SXT, GM, TE, RA	*aac* (*6*′)-*aph* (*2*^″^), *ermC*
23.	t1258	II	Trachea	III	*tst*, *hla*, *seb*	FOX, ERY, CD, SXT, GM, TE, RA	*aac* (*6*′)-*aph* (*2*^″^), *aph3*, *ermA*
24.	t1258	I	Trachea	I	*pvl*, *hla*, *sea*	FOX, ERY, CD, TE, RA	*aac* (*6*′)-*aph* (*2*^″^), *aph3*, *ermC*
25.	t1360	I, II	Wound	III	*hla*, *sea*	FOX, ERY, CD, SXT, RA	*aac* (*6*′)-*aph* (*2*^″^), *ermC*
26.	t1360	III	Wound	IX	*hla*, *sea*, *sec*	FOX, ERY, CD, SXT, GM, RA	*aac* (*6*′)-*aph* (*2*^″^), *aph3*, *ermC*
27.	t131	II	Wound	III	*pvl*, *hla*, *seb*	FOX, ERY, CD, SXT, GM, TE, RA	*aac* (*6*′)-*aph* (*2*^″^), *ermC*
28.	t5163	II	Trachea	III	*pvl*, *hla*, *seb*	FOX, ERY, CD, GM, TE	*aac* (*6*′)-*aph* (*2*^″^), *ermC*
29.	t5163	I	Wound	III	*hla*	FOX, ERY, CD, TE	*ant4*, *ermC*
30.	t7685	II	Urine	III	*tst*, *hla*	FOX, ERY, CD, SXT, GM, TE	*aac* (*6*′)-*aph* (*2*^″^), *aph3*, *ermC*
31.	t7685	III	Blood	III	*tst*, *hla*, *sea*	FOX, ERY, CD, SXT, GM	*aac* (*6*′)-*aph* (*2*^″^), *ant4*, *ermC*
32.	t1587	II	Nasal	III	*hla*, *sea*	FOX, ERY, CD, GM, TE	*aac* (*6*′)-*aph* (*2*^″^), *aph3*, *ermB*
33.	t1403	II	Nasal	V	*tst*, *hla*, *sea*, *seb*	FOX, ERY, SXT, GM	*aac* (*6*′)-*aph* (*2*^″^), *ant4*
34.	t15871	II	Trachea	III	*pvl*, *hla*	FOX, ERY, CD, GM, TE, RA	*ant4*, *ermC*
35.	t159	I	Trachea	III	*hla*, *sea*, *seb*, *sec*	FOX, ERY, CD, GM, TE, RA	*ant4*, *ermC*
36.	t2104	II	Wound	III	*hla*, *sea*	FOX, ERY, CD, TE, RA	*aac* (*6*′)-*aph* (*2*^″^), *ermC*
37.	t275	II	Nasal	III	*pvl*, *hla*	FOX	*—*
38.	t223	II	Nasal	V	*tst*, *seb*	FOX, ERY, CD, TE	*ant4*, *ermA*
39.	t2457	II	Blood	III	*pvl*, *hly*, *seb*	FOX, ERY, CD, GM, TE, RA	*aac* (*6*′)-*aph* (*2*^″^), *aph3*
40.	t314	II	Wound	III	*hla*	FOX, ERY, CD, GM, TE, RA	*aac* (*6*′)-*aph* (*2*^″^), *ermC*
41.	t2976	II	Urine	III	*pvl*, *tst*, *hla*, *sea*	FOX, ERY, CD, SXT, GM, TE, RA	*aac* (*6*′)-*aph* (*2*^″^), *aph3*, *ermA*
42.	t3182	I	Blood	III	*pvl*, *tst*, *hla*, *sea*	FOX, ERY, CD, GM, TE, RA	*ant4*, *ermC*
43.	t3236	III	Nasal	III	*pvl*, *hla*	FOX, ERY, CD, GM, TE	*aac* (*6*′)-*aph* (*2*^″^), *ermC*
44.	t3204	II	Wound	II	*hla*, *seb*	FOX, ERY, CD, SXT, GM, TE, RA	*aac* (*6*′)-*aph* (*2*^″^), *ant4*
45.	t3204	I	Nasal	III	*hla*, *sea*	FOX, ERY, CD, GM	*ermC*, *ermB*
46.	t325	I	Trachea	III	*hla*	FOX, ERY, CD, GM, TE, RA	*aac* (*6*′)-*aph* (*2*^″^), *ant4*, *ermC*
47.	t325	II	Wound	III	*pvl*, *tst*	FOX, ERY, GM	*ant4*, *ermA*
48.	t325	IV	Wound	III	*hla*, *sea*	FOX, ERY, CD, GM, TE	*aph3*, *ant4*, *ermA*
49.	t4679	II	Nasal	III	*pvl*, *tst*, *hla*, *sea*, *seb*	FOX, ERY, CD, GM, RA	*aac* (*6*′)-*aph* (*2*^″^), *ermB*
50.	t3769	II	Synovial	I	*pvl*, *hla*, *sea*	FOX, ERY, CD, RA	*ermA*, *ermB*
51.	t459	III	Blood	V	*hla*, *sea*	FOX, ERY, CD, TE, RA	*ermA*, *ermB*
52.	t5593	II	Urine	IX	*hla*	FOX, ERY, CD, SXT, GM, TE	*ant4*, *ermC*
53.	t692	III	Nasal	III	*hla*	FOX, ERY, CD, SXT, GM, TE, RA	*ant4*, *ermA*
54.	t790	II	Nasal		*hla*, *sea*	FOX, ERY, CD, SXT, GM, TE	*aac* (*6*′)-*aph* (*2*^″^), *ermC*
55.	t459	II	Nasal	III	*hla*, *sea*	FOX, ERY, CD, GM	*ant4*, *ermB*

## Data Availability

The authors confirm that the data supporting the findings of this study are available within the article.
